# Effects of oxygen levels and a *Lactobacillus plantarum* strain on mortality and immune response of chickens at high altitude

**DOI:** 10.1038/s41598-019-52514-w

**Published:** 2019-11-05

**Authors:** Lihong Wang, Guanhua Fu, Suozhu Liu, Long Li, Xin Zhao

**Affiliations:** 1 College of Animal Science and Technology, Northwest A & F University, Yangling, Shaanxi 712100 China; 2 College of Animal Science, Tibet Agriculture and Animal Husbandry College, Linzhi, Tibet 860000 China; 3 Department of Animal Science, McGill University, Montreal, QC H9X 3V9 Canada

**Keywords:** Immunology, Physiology

## Abstract

Chickens reared in high altitude regions suffer from a high mortality, possibly due to poor immune responses induced by hypoxia. This experiment was conducted to evaluate whether increasing the oxygen level or administration of a probiotic could improve mortality and immune response of chickens at high altitude (2,986 m above the sea level). One-d-old chickens were randomly allocated to 1 of 6 treatments in a 2 × 3 factorial arrangement. The first factor was the oxygen level (low and high), while the second factor was the diet (basal diet, basal diet containing aureomycin, and basal diet plus *L. plantarum*). Increasing the oxygen level significantly reduced the mortality and improved immune responses. The levels of IgA, IgG, IL-10 and anti-BSA antibodies were significantly higher, while IL-1β, LITAF were significantly lower in chickens reared in the high-oxygen room. In the low-oxygen room, *L. plantarum* significantly decreased the mortality of chickens compared with the other 2 groups. Moreover, *L. plantarum* significantly increased the levels of IgA, anti-BSA antibodies, IL-10 and decreased IL-1β, LITAF compared with the control group. These results demonstrated that increasing oxygen level or administration of *L. plantarum* can improve health status of chickens in high altitude regions.

## Introduction

More commercial broiler chickens are being raised in Tibet, China to meet the requirement for increased consumption. However, the mortality rates are high, causing great economic losses to farmers. Our previous study showed that the mortality of commercial broiler chickens reared in Linzhi city (average 2,986 m above the sea level) was about 30% during a 42 days rearing period, with approximately 50% of the death occurring during the first 14 days^1^. In that study, all dead birds during the experimental period were necropsied to identify ascites-related mortality or non-ascites induced mortality. Non-ascites induced mortality accounted for 83.3% of the total mortality and ascites induced mortality accounted for 16.7% of the total mortality during the first 2 weeks^1^. In addition, the production performance of chickens reared at high altitude is low^1,2^. Therefore, improving the production performance and decreasing the mortality are crucial for broiler production in Tibet and other high altitude regions.

High altitude hypoxia refers to a state in which oxygen supply is insufficient due to a low partial pressure of oxygen in air. Thus, increasing oxygen levels of chicken houses may decrease mortality and improve immune responses of broilers reared in high altitude regions. Hypoxia can influence the immune response of animals. Hypoxia could affect the differentiation and functions of immune cells, such as T and B lymphocytes^3,4^, thereby influencing the levels of immunoglobulins and cytokines. Kleessen *et al*.^5^ found that the levels of IgA and IgM in blood had a tendency to decrease in German mountaineers during expedition in Nepalese Himalayas. On the other hand, Chohan *et al*.^6^ found that the levels of IgG and IgA were significantly higher in high altitude natives and the sea level residents at high altitude for 2 years compared with the sea level residents. The levels of cytokines can also be influenced by hypoxia, thereby disrupting the balance between proinflammatory and anti-inflammatory cytokines. Hartmann *et al*.^7^ found that the levels of pro-inflammatory cytokine IL-6, the inflammatory marker IL-1ra, and C-reactive protein were all significantly increased when 12 healthy people stayed at 3,458 meters above the sea level for 68 hours. Yang *et al*.^8^ found that hypoxia significantly increased the secretion of IL-4 and decreased the secretion of IFN-γ in naïve CD4^+^ T cells. Nevertheless, the effects of hypobaric hypoxia on immunological changes of broiler chickens, which may be associated with high mortality of starter phase of chickens reared at high altitude, has not been reported. Therefore, one objective of this study was to evaluate whether increasing oxygen levels had beneficial effects on broilers reared at high altitude.

Probiotics can improve production performance^9^, maintain the balance of gut microbiota^10^, modulate the immune responses^11,12^, and protect chickens from pathogenic infections^13,14^. However, the effects of probiotics on mortality, and immunity function of broiler chickens under the hypobaric hypoxic condition are still unknown. So, another objective of this study was to determine the effects of a potential probiotic on broilers reared at high altitude.

## Results

### Blood parameters

As shown in Table 1, increasing the oxygen level did not influence the red blood cell (RBC) counts, hemoglobin (HGB) levels and hematocrit (HCT) values at day 7 (*P* > 0.05). However, chickens reared in the low-oxygen room exhibited significantly higher (*P* < 0.05) RBC counts, HGB levels and HCT values than the chickens reared in the high-oxygen room at day 14. The probiotic had no effect (*P* > 0.05) on RBC counts, HGB levels and HCT values.

**Table 1:** Effects of different treatments on the blood parameters of chickens.

Items^1^	Day 7			Day 14		
	RBC, 10^12^/L	HGB, g/L	HCT, %	RBC, 10^12^/L	HGB, g/L	HCT, %
**Treatment**						
**Low-oxygen**						
CON	1.69	71.17	25.95	2.40	126.00	30.03
AUR	1.66	72.00	26.72	2.42	130.83	31.72
PRO	1.68	74.67	27.44	2.36	128.67	30.50
**High-oxygen**						
CON	1.65	70.83	26.78	2.27	120.17	27.87
AUR	1.67	69.50	26.99	2.22	123.67	28.12
PRO	1.69	73.67	27.41	2.25	118.00	28.32
SEM^2^	0.03	1.05	0.31	0.03	1.60	0.51
**Main effect oxygen (n = 18)**						
Low-oxygen	1.67	72.61	26.70	2.39^a^	128.50^a^	30.75^a^
High-oxygen	1.68	71.33	27.06	2.25^b^	120.61^b^	28.10^b^
**Main effect probiotic (n = 12)**						
CON	1.67	71.00	26.37	2.34	123.08	28.95
AUR	1.66	70.75	26.86	2.32	127.25	29.92
PRO	1.69	74.17	27.42	2.31	123.33	29.41
**P-values**						
Oxygen	0.939	0.546	0.569	0.033	0.019	0.015
Probiotic	0.930	0.344	0.388	0.922	0.497	0.745
Oxygen × Probiotic	0.908	0.931	0.848	0.847	0.817	0.809

^1^RBC: red blood cell counts; HGB: hemoglobin level; HCT: hematocrit values. High-oxygen: chickens were reared in a room with oxygen percentages between 25.8% and 26.5%; Low-oxygen: chickens were reared in a room with oxygen percentages between 20.7% and 21.4%. CON: chickens were fed basal diet; AUR: chickens were fed basal diet supplemented with aureomycin (50 mg/kg diet); PRO: chickens were fed basal diet and inoculated with *L. plantarum* (10^9^ CFU/chicken) by gavage on day 1. ^2^SEM: standard error of a mean (n = 6 pens per treatment group). ^a,b^Different superscripts indicate significant differences (*P* < 0.05).

### Production performance

The body weight (BW) and average daily feed intake (ADFI) of broilers in the high-oxygen room were significantly higher (*P* < 0.05) on day 14 than those in the low-oxygen room. However, the probiotic had no effect (*P* > 0.05) on the BW and ADFI of broilers (Table 2). Both increasing oxygen levels and administration of the probiotic had no effect (*P* > 0.05) on the feed conversion ratio (FCR).

**Table 2:** Effects of different treatments on production performance and relative weight of immune organs.

Items^1^	Performance (d 1–14)			Relative weight of immune organs (d 14), g/kg		
	BW(d14), g	ADFI, g	FCR	Thymus	Spleen	Fabricius
**Treatment**						
**Low-oxygen**						
CON	165.33	18.30	2.16	1.03	1.13	1.39
AUR	168.90	18.50	2.12	1.01	1.18	1.22
PRO	171.07	19.68	2.22	1.45	1.22	1.41
**High-oxygen**						
CON	192.80	22.02	2.12	1.60	1.49	1.38
AUR	196.76	22.90	2.14	1.65	1.19	1.44
PRO	193.22	22.50	2.15	1.99	1.55	1.70
SEM^2^	2.01	0.22	0.03	0.07	0.05	0.07
**Main effect oxygen (n** = **18)**						
Low-oxygen	168.43^b^	18.83^b^	2.16	1.16^b^	1.18^b^	1.34
High-oxygen	194.26^a^	22.47^a^	2.14	1.74^a^	1.41^a^	1.51
**Main effect probiotic (n** = **12)**						
CON	179.07	20.16	2.14	1.31^b^	1.31	1.39
AUR	182.83	20.70	2.13	1.33^b^	1.18	1.33
PRO	182.14	21.09	2.19	1.72^a^	1.39	1.55
**P-values**						
Oxygen	0.001	0.001	0.663	0.001	0.041	0.236
Probiotic	0.721	0.227	0. 667	0.021	0.312	0.393
Oxygen × Probiotic	0.813	0.340	0.789	0.937	0.385	0.657

^1^BW: body weight; ADFI: average daily feed intake; FCR: feed conversion ratio. High-oxygen: chickens were reared in a room with oxygen percentages between 25.8% and 26.5%; Low-oxygen: chickens were reared in a room with oxygen percentages between 20.7% and 21.4%; CON: chickens were fed basal diet; AUR: chickens were fed basal diet supplemented with aureomycin (50 mg/kg diet); PRO: chickens were fed basal diet and inoculated with *L. plantarum* (10^9^ CFU/chicken) by gavage on day 1. ^2^SEM: standard error of a mean (n = 6 pens per treatment group). ^a,b^Different superscripts indicate significant differences (*P* < 0.05).

### The relative weight of immune organ

As shown in Table 2, the relative weights of thymus and spleen were significantly increased (*P* < 0.05) by the high-oxygen treatment. Similarly, the chickens in the probiotic (PRO) group had significantly higher (*P* < 0.05) relative weight of thymus than those in the other 2 groups.

### Mortality

**Figure 1:**
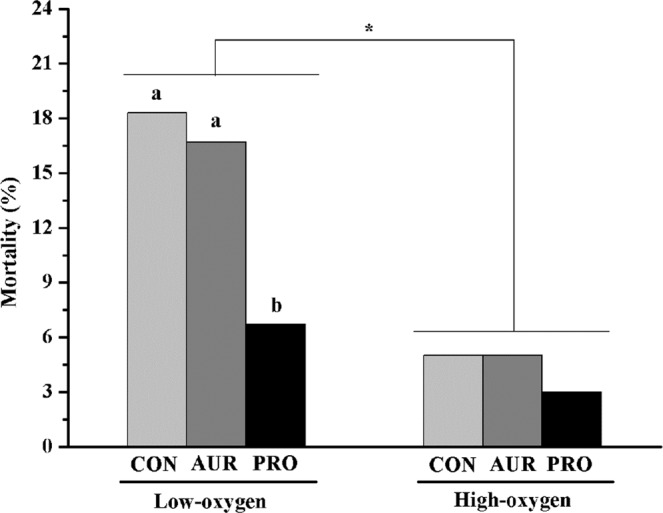
Effects of different treatments on mortality of chickens. High-oxygen: chickens were reared in a room with oxygen concentrations between 25.8% and 26.5%; Low-oxygen: chickens were reared in a room with oxygen concentrations between 20.7% and 21.4%. CON: chickens were fed basal diet; AUR: chickens were fed basal diet supplemented with aureomycin (50 mg/kg diet); PRO: chickens were fed basal diet and inoculated with *L. plantarum* (10^9^ CFU/chicken) by gavage on day 1. *Indicates significant differences between high-oxygen room and low-oxygen room (*P* < 0.05). ^a,b^Different letters indicate significant dietary differences within the same room (*P* < 0.05).

The mortality of chickens reared in the high-oxygen room was significant lower (*P* < 0.05) than the low-oxygen room (Fig. 1). In the low-oxygen room, the probiotic treatment significantly decreased (*P* < 0.05) the mortality of chickens compared with the control (CON) and the aureomycin (AUR) groups. However, the probiotic had no effect (*P* > 0.05) on the mortality of chickens reared in the high-oxygen room.

### Plasma immunoglobulins and antibody responses to bovine serum albumin (BSA)

Chickens reared in the high-oxygen room had significantly higher levels of IgA levels on day 7 and both IgA and IgG on day 14 than those in the low-oxygen room (*P* < 0.05) (Table 3). Compared with the CON and AUR groups, the probiotic significantly increased (*P* < 0.05) the levels of IgA, but not IgG and IgM, on day 7 and day 14.

**Table 3:** Effects of different treatments on the plasma immunoglobulin of chickens.

Items^1^	Day 7			Day 14		
	IgA, ng/ml	IgG, ng/ml	IgM, ng/ml	IgA, ng/ml	IgG, ng/ml	IgM, ng/ml
**Treatment**						
**Low-oxygen**						
CON	98.99	464.77	181.77	213.96^b^	611.10	336.90
AUR	100.19	466.72	189.65	228.74^b^	630.79	358.13
PRO	112.47	455.29	187.39	257.83^a^	627.58	363.56
**High-oxygen**						
CON	109.14	488.90	192.19	263.86^b^	731.87	363.10
AUR	107.33	505.33	190.70	270.62^b^	721.04	380.10
PRO	116.43	472.53	195.02	303.93^a^	720.34	379.77
SEM^2^	1.75	7.96	4.85	4.87	12.00	5.33
**Main effect oxygen (n** = **18)**						
Low-oxygen	103.88	462.26	186.27	233.51^b^	623.16^b^	350.00
High-oxygen	110.97	488.92	192.64	279.14^a^	724.41^a^	374.32
**Main effect probiotic (n** = **12)**						
CON	104.07^b^	476.84	186.98	240.14^b^	671.48	350.00
AUR	103.76^b^	486.03	190.18	249.68^b^	675.92	369.11
PRO	114.45^a^	463.91	191.21	280.88^a^	673.96	371.67
**P-values**						
Oxygen	0.050	0.110	0.516	0.001	0.001	0.059
Probiotic	0.026	0.510	0. 934	0.010	0.989	0.226
Oxygen × Probiotic	0.775	0.850	0.926	0.962	0.856	0.971

^1^High-oxygen: chickens were reared in a room with oxygen percentages between 25.8% and 26.5%; Low-oxygen: chickens were reared in a room with oxygen percentages between 20.7% and 21.4%. CON: chickens were fed basal diet; AUR: chickens were fed basal diet supplemented with aureomycin (50 mg/kg diet); PRO: chickens were fed basal diet and inoculated with *L. plantarum* (10^9^ CFU/chicken) by gavage on day 1. ^2^SEM: standard error of a mean (n = 6 pens per treatment group). ^a,b^Different superscripts indicate significant differences (*P* < 0.05).

**Figure 2:**
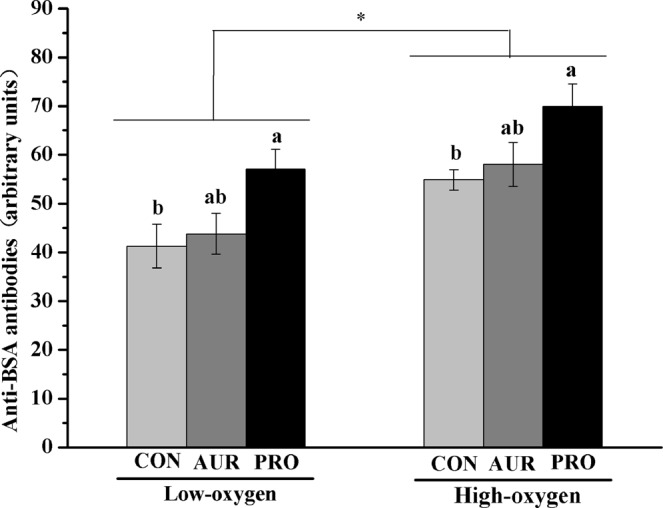
Effects of different treatments on anti-BSA antibodies of chickens. High-oxygen: chickens were reared in a room with oxygen concentrations between 25.8% and 26.5%; Low-oxygen: chickens were reared in a room with oxygen concentrations between 20.7% and 21.4%. CON: chickens were fed basal diet; AUR: chickens were fed basal diet supplemented with aureomycin (50 mg/kg diet); PRO: chickens were fed basal diet and inoculated with *L. plantarum* (10^9^ CFU/chicken) by gavage on day 1. *Indicates significant differences between the high-oxygen room and the low-oxygen room (*P* < 0.05, n = 24). ^a,b^Different letters indicate significant dietary differences within the same room (*P* < 0.05, n = 6).

To evaluate the special antibody response, the level of antibodies against BSA in plasma of chickens was assessed. Plasma of broilers in the high-oxygen room had a significantly higher (*P* < 0.05) level of IgG antibodies against BSA compared with the low-oxygen room (Fig. 2). In addition, *L. plantarum* significantly increased (*P* < 0.05) anti-BSA IgG antibodies compared with the CON and AUR groups in both rooms.

### Cytokines

To evaluate the effects of oxygen levels and *L. plantarum* treatments on cytokines production, ELISA was performed to examine levels of IL-1β, LITAF, IL-6, IL-10 and IL-12 in plasma. As shown in Table 4, on day 7, increasing oxygen levels significantly decreased the levels of IL-1β, LITAF, and IL-6 (*P* < 0.05) and increased (*P* < 0.05) the level of IL-10 compared with chickens in the low-oxygen room. There was no significant difference in the level of IL-12 between 2 rooms. *L. plantarum* significantly increased (*P* < 0.05) the level of IL-10 and decreased the level of LITAF (*P* < 0.05) compared with the CON group, but had no effect (*P* > 0.05) on the levels of IL-1β, IL-6 and IL-12. Aureomycin significantly increased the level of IL-10 (*P* < 0.05), but had no effect on the other cytokines.

**Table 4:** Effects of different treatments on the plasma cytokines of broiler chickens.

Items^1^	Day 7					Day 14				
	IL-6, pg/ml	IL-1β, ng/ml	LITAF, ng/ml	IL-10, pg/ml	IL-12, pg/ml	IL-6, pg/ml	IL-1β, ng/ml	LITAF, ng/ml	IL-10, pg/ml	IL-12, pg/ml
**Treatment**										
**Low-oxygen**										
CON	106.32	4.43	2.57	450.68^b^	102.29	343.05	5.78^a^	3.52^a^	493.86	195.61
AUR	109.20	4.42	2.39	472.36^b^	90.19	310.04	4.55^ab^	2.93^ab^	532.62	185.08
PRO	116.04	4.35	1.84	554.66^a^	90.27	302.55	4.30^b^	2.59^b^	588.50	184.29
**High-oxygen**										
CON	104.95	2.85	1.80	487.23^b^	91.15	308.94	4.36^a^	2.82^a^	605.37	188.96
AUR	103.80	2.90	1.34	602.50^ab^	93.53	325.91	3.19^b^	2.03^b^	609.21	168.02
PRO	104.45	3.04	1.34	617.72^a^	91.05	329.82	3.26^b^	1.92^b^	724.66	160.01
SEM^2^	1.51	0.20	0.07	13.25	3.03	9.95	0.15	0.11	16.84	5.26
**Main effect oxygen (n = 18)**										
Low-oxygen	110.52	4.40^a^	2.27^a^	492.57^b^	94.24	318.55	4.88^a^	3.03^a^	538.33^b^	188.32
High-oxygen	104.40	2.93^b^	1.49^b^	569.15^a^	91.91	321.56	3.60^b^	2.25^b^	646.41^a^	172.34
**Main effect Probiotic (n = 12)**										
CON	105.63	3.64	2.19^a^	468.96^b^	96.72	326.00	5.07^a^	3.19^a^	549.71^b^	192.28
AUR	106.50	3.66	1.87^b^	537.43^a^	91.86	317.97	3.87^b^	2.48^b^	570.91^ab^	176.55
PRO	110.24	3.70	1.59^b^	586.19^a^	90.66	316.19	3.78^b^	2.25^b^	656.58^a^	172.17
**P-values**										
Oxygen	0.050	0.001	0.019	0.008	0.700	0.881	0.001	0.003	0.005	0.150
Probiotic	0.416	0.992	0.012	0.006	0.713	0.921	0.007	0.011	0.035	0.316
Oxygen × Probiotic	0.388	0.948	0.386	0.313	0.619	0.460	0.864	0.902	0.757	0.807

^1^LITAF: Lipopolysaccharide-induced TNF-α factor; High-oxygen: chickens were reared in a room with oxygen percentages between 25.8% and 26.5%; Low-oxygen: chickens were reared in a room with oxygen percentages between 20.7% and 21.4%. CON: chickens were fed basal diet; AUR: chickens were fed basal diet supplemented with aureomycin (50 mg/kg diet); PRO: chickens were fed basal diet and inoculated with *L. plantarum* (10^9^ CFU/chicken) by gavage on day 1. ^2^SEM: standard error of a mean (n = 6 pens per treatment group). ^a,b^Different superscripts indicate significant differences (*P* < 0.05).

On day 14, no significant differences in the levels of IL-6 and IL-12 was observed among all groups. However, the levels of IL-1β and LITAF for the chickens in the low-oxygen room were higher (*P* < 0.05) than chickens in the high-oxygen room. Increasing oxygen levels caused the increase (*P* < 0.05) of IL-10 significantly. The levels of IL-1β and LITAF were decreased (*P* < 0.05) by the *L. plantarum* treatment. *L. plantarum* significantly increased the level of IL-10 (*P* < 0.05). Aureomycin decreased (*P* < 0.05) the levels of IL-1β and LITAF, but had no effect on the other cytokines.

## Discussion

To the best of our knowledge, this study is the first to evaluate the effect of hypoxia on broilers by increasing the oxygen level at high altitude. In the past, researchers usually studied the effect of hypoxia on broilers by simulated high altitude in hypobaric chambers^15,16^. Since only two rooms were used in this study, all efforts were made to keep temperature, humidity, management, ventilation and sterilization measures same in these 2 rooms. So, the only major difference between the high-oxygen room and the low-oxygen room was oxygen levels. The interesting result from the present study was that increasing the oxygen level significantly improved the production performance, evidenced by higher BW of the broilers reared in the high-oxygen room at day 14. Yersin *et al*.^17^ demonstrated that broilers reared at simulated high altitude (2,133 meters above sea level) had significantly lower body weights compared with chickens reared at natural low altitude (100 m above sea level) at day 14 and day 35. Li *et al*.^1^ found that the body weight of chickens reared in high altitude (Linzhi, China) at d 42 was significantly lower than the expected growth performance at 42 d based on NRC^18^. High altitude hypoxia caused severe intestinal mucosal injury^19^ and depressed gut development^1^. These negative effects can result in reduced absorption efficiency for nutrients^20^. In addition, animals may be anorectic under the hypoxic condition. ADFI of broilers reared in the low-oxygen room were lower than broilers reared in the high-oxygen room. Increasing oxygen levels also decreased the mortality of chickens significantly. In our previous study performed in the same location, the mortality of chickens during the starter phase (first 14 days) account for half of the total mortality during the whole 42 days experimental period^1^. The price of an oxygen generator is about RMB 3,000, and the power of an oxygen generator was less than 400 W. Therefore, increasing oxygen levels of chicken houses by an oxygen concentrator may be a cost-effective measure to improve production performance of broilers reared in high altitude regions.

Hematological changes are important physiological responses to the hypobaric hypoxic condition. In the current study, hematological changes were obvious. The RBC counts, HGB level and HCT of broiler chickens reared in the low-oxygen room were all higher than the high-oxygen room on day 14. Our result is also supported by a previous study by Yersin *et al*.^17^, who showed that chickens reared at a simulated high altitude (2,133 meters above the sea level) had higher RBC counts, HGB level and HCT value than chickens reared at a low altitude (100 meters above the sea level). By increasing RBC counts and HGB levels, chickens increase the oxygen-carrying capacity by a compensatory mechanism to the hypobaric hypoxic condition.

The high mortality of chickens reared in low-oxygen room may be associated with the change of immune responses. The relative weights of lymphoid organs are often used to predict the immune status of broiler chickens^21^. In this study, chickens reared in the low-oxygen room had significantly lower relative weight of thymus and spleen compared with chickens reared in the high-oxygen room. In addition, immunoglobulins play an important role in immune function of animals. In this study, the levels of IgA and IgG in chickens reared in the low-oxygen room were significantly lower than chickens reared in the high-oxygen room on day 14. In addition to non-specific antibody responses, hypoxia could also influence the specific immune response. The level of anti-BSA IgG of chickens reared in the low-oxygen was lower than chickens in the high-oxygen room, suggesting that increasing oxygen levels could improve the immune responses of broilers in high altitude regions. Hypoxia functioning as a danger signal for the immune system could also influence the synthesis of cytokines. In the present study, chickens reared in the low-oxygen level room had significantly higher levels of IL-1β and LITAF than chickens in the high-oxygen room at day 7 and day 14. In addition, hypoxia also increased the level of IL-6 and decreased the level of IL-10 at day 7. LITAF, IL-1β, and IL-6 are proinflammatory cytokines that involved in inflammatory responses. IL-10 is an anti-inflammatory cytokine and responsible for downregulation inflammatory responses. The increase of LITAF, IL-1β, and IL-6 and the decrease of IL-10 indicated an early inflammatory response in chickens reared in the low-oxygen room^22^.

To our best knowledge, this research is also the first to study the effects of the probiotics on the broilers reared in high altitude. During the experiment period, *L. plantarum* had no effect on production performance of broilers in both high-oxygen room and low-oxygen room. Although many studies have suggested the beneficial effects of probiotics in improving broiler performance, the growth-promoting efficacy of probiotics is more effective during the grower phase (from day 15 to day 28) and the finisher phase (from day 29 to day 42) rather than during the starter phase (from day 1 to day 14)^10,23^. Zhang *et al*.^23^ found that a diet supplemented with a mixture of *L. acidophilus, B. subtilis*, and *C. butyricum* (1 × 10^5^ and 2 × 10^5^ CFU/kg of diet) significantly improved the body weight gain during day 22 to day 35, not during day 1 to day21. Similarly, Mountzouris *et al*.^10^ found that a diet supplemented with 5-bacterial species probiotic product (10^8^ CFU probiotics/kg diet) significantly increased growth performance of broiler chickens during the grower and finisher phases, but had no effect on broiler production performance for the starter phase (day 1 to day14). In the present study, the focus was on the first 14 days because the young chicks were more sensitive to hypoxia than adult chickens, in terms of the mortality rate^1^.

Hypoxia is a challenge to animals. Cataldi *et al*.^24^ and Wang *et al*.^25^ reported that hypoxia could induce the secretion of large quantity of ROS which was involved in oxygen stress. In the present study, probiotic decreased mortality of chickens reared in low-oxygen room. However, probiotic had no effect on the mortality of chickens reared in the high-oxygen room. This was probably due to the fact that a probiotic is more effective when chickens are under stress. For example, Zulkifli *et al*.^26^ found that probiotics could increase the antibody response against the Newcastle disease vaccine only after chickens were subjected to heat stress. The *L. plantarum* treatment increased the level of IgA on day 14. In addition, chickens in the PRO group had stronger anti-BSA IgG responses. Finally, *L. plantarum* reduced the levels of proinflammatory cytokines and increased the level of anti-inflammatory cytokine. All these results suggest that *L. plantarum* improved the immune functions of chickens in high altitude regions. Our results were in accordance with previous results by others. For example, Zhang *et al*.^23^ found that the levels of serum IgA were increased by administration of multistrain probiotics. Wang *et al*.^27^ also demonstrated that a novel *L. plantarum* strain P-8 increased IgA^+^ lymphocytes in the jejunum and Peyer’s patches. Rajput *et al*.^12^ found that probiotics could increase the IgA positive cells significantly in the jejunum of broilers. This may be associated with the increase of the level of IgA. Brisbin *et al*.^28^ reported that *L. salivarius*-treated broilers had significantly more serum antibody to sheep red blood cell (SRBC) and keyhole limpet hemocyanin (KLH) than birds that were not treated with probiotics after they were immunized with SRBC and KLH. Feng *et al*.^13^ and Chen *et al*.^14^ found that probiotics could modulate the expression of proinflammatory and anti-inflammatory cytokines.

In conclusion, the results of the present study indicated that increasing oxygen levels of broiler houses can decrease mortality and modulate the immune response of broilers reared at high altitude during the starter phase. It may be a cost-effective measure to improve the production performance and decrease mortality of broilers in high altitude regions. In addition, *L. plantarum* can modulate the immune responses of broiler chickens reared at high altitude and it could act as an immunomodulator for improving the immune responses of broilers.

## Methods

### Bacterial strain and growth conditions

Probiotic used in this study was isolated from intestine of a healthy Tibet local chicken, and identified as *Lactobacillus plantarum* (*L. plantarum*) by 16S rRNA sequencing. *L. plantarum* were cultured in the de Man, Rogosa and Sharp broth, grown at 37 °C until stationary growth phase. All bacteria were harvested by centrifugation (6,000 × g for 15 min) at the beginning of stationary growth phase. Pelleted bacteria were then washed 3 times using sterile phosphate-buffered saline (PBS) and diluted in PBS. One hundred μl of *L. plantarum* suspension was serially diluted in sterile PBS, then 100 μl diluted suspension was plated onto the de Man, Rogosa and Sharp agar to enumerate colonies after incubation at 37 °C for 24 h. *L. plantarum* counts were expressed as CFU per milliliter.

### Chickens and housing

A total of 360 newly hatched 1-d-old healthy Arbor Acres broilers of mixed sex were purchased from a commercial hatchery at Chengdu (average altitude 500 m above the sea level), then transported to the experimental farm of Tibet Agriculture and Animal Husbandry College (average altitude 2,986 m above the sea level) by air for this study. The chicks were randomly allotted to 6 treatments, taking the sex into the consideration. Same numbers of males and females were assigned in each pen. Each treatment consisted of 6 replicate pens with 10 chicks in each pen. The chicks were raised in 3-layer metal cages, the average stocking density was 15 birds per square meter. The room temperature was maintained at 33 °C for the first week and 28 °C for the second week. The chicks were allowed ad libitum access to the experimental diets and water. Chicks were subjected to 23 h of light and 1 h of dark throughout the trial. This research was approved by the Northwest Agriculture and Forestry (A&F) University Institutional Animal Care and Use Committee protocol number (NWAFAC2826), and all experimental protocols used in this study were in accordance with the relevant guidelines approved by the Northwest Agriculture and Forestry (A&F) University Institutional Animal Care. The institutional safety procedures were followed.

### Experimental design

The experiment was in a 2 × 3 factorial design and last 22 days. The first factor contained 2 levels: low-oxygen concentration (20.7% to 21.4%) and high-oxygen concentration (25.8% to 26.5%). The altitude will not affect the oxygen concentration in the air. However, the rising of the altitude decreases the atmospheric pressure and the oxygen level which equals to atmospheric pressure *oxygen concentrations. By increasing the oxygen concentration in a room, the oxygen level was increased in the air. The atmospheric pressure in Linzhi is 519 mm Hg. High-oxygen level was achieved by supplementing a room with oxygen (3 L/min, purity 93% ± 3%) using an oxygen concentrator (Yuwell, 8F-3W, Jiangsu Yuyue medical equipment & supply Co., Ltd, China). The oxygen concentrator separates oxygen from air. The machine was put outside of the room and oxygen was imported into the room by a tube. Oxygen concentrations were measured using an oxygen monitor (AS8901, The Smart Sensor, Dongguan, China). All other factors including temperature, humidity, management, ventilation and sterilization measures were kept same for the two rooms. The second factor was diet, including a control diet, an aureomycin supplemented diet and a control diet supplemented with a probiotic. The birds in the control group (CON) and the probiotic group (PRO) were fed the basal diet alone. The birds in the aureomycin group (AUR) were fed the basal diet containing aureomycin at 50 mg/kg of diet. The chicks in the PRO group were inoculated via oral gavage with 0.2 ml PBS containing 10^9^ CFU *L. plantarum* on the day of arriving at the experimental houses. PBS was used as a placebo for the other 2 groups. The basal diet was a corn-soybean starter broiler diet (12.6 MJ metabolizable energy/kg of diet, 220 g/kg crude protein) formulated without antibiotics or coccidiostats. The nutrient levels met or exceeded critical levels of nutrients recommended by the National Research Council^18^.

### Immunization and oral antigen administration

In order to measure specific antibody responses, broiler chickens were immunized orally with BSA (Sigma-Aldrich, Zwijndrecht, The Netherlands). Our preliminary experiment indicated that the BSA-antibodies were undetectable in serum if chickens were orally administrated with BSA before day 8. The level of BSA-antibodies 10 days after the last oral immunization was high if chickens were orally administrated with BSA between day 8 and day 12. Our observation was also supported by the results from Bar-Shira *et al*.^29^. Therefore, 2 broiler chickens of every replicate were randomly chosen to be immunized with BSA (5 mg/day per chicken) for 5 days from 8-day-old to 12-day-old, BSA was dissolved in sterile water, and another 2 broiler chickens were inoculated with sterile water as the control. Oral immunization was performed by gently placing a blunt-tipped feeding needle above the tongue, and slowly dripping the solution into the pharynx, thus allowing the chickens to voluntarily swallow the solution. Ten days after the last immunization, blood samples were collected from the jugular vein. Samples were centrifuged for 10 min at 3,000 × g, and plasma was harvested and stored at −20 °C.

### Production performance

Body weight for chickens in each pen and feed intake (FI) were obtained on day14. The FCR, defined as FI divided by the weight gain, was calculated for the first 2 weeks of the experiment. Mortality was recorded daily.

### The relative weight of immune organ

On day 14, 1 broiler chicken per replicate was randomly selected from every group and euthanized via cervical dislocation immediately after weighing. The immune organs (spleen, thymus and bursa of Fabricius) were harvested and weighted and the relative weight of organ was calculated as the ratio of an organ weight divided by the body weight (g/kg).

### Analyses of blood samples

Blood samples were collected from the jugular vein on day 7 and day 14. A part of blood sample was used for measuring RBC counts, HGB levels and HCT values by an automatic blood analyzer (XFA6000, Pulang Company, Nanjing, China) which had been standardized for analyses of chicken blood.

The remaining blood samples were centrifuged for 10 min at 3,000 × g, and plasma were harvested and stored at −20 °C for analyses of IgA, IgG, IgM and cytokines. The levels of plasma IgA, IgG and IgM were measured by a sandwich enzyme-linked immunosorbent assay (ELISA) using chicken-specific IgA, IgG and IgM ELISA quantitation kits (Elabscience Biotechnology, Wuhan, China), following the instruction of the manufacturer. The levels of IgA, IgM, and IgG were determined using standard curves constructed from respective immunoglobulin standards and were expressed as nanogram of IgA, IgM, or IgG per milliliter of plasma.

### Assays for anti-BSA antibodies

Anti-BSA antibody levels were determined by ELISA. Briefly, 96-well microplates were coated with BSA solution and incubated overnight at 4 °C. Blocking was performed with skim milk. The plates were washed before adding plasma samples (diluted to a proper concentration) to each well. Then, peroxidase labeled goat anti-chicken IgG was added to each well. Finally, bound antibodies were detected by 3,3′,5,5′-Tetramethylbenzidine followed by stop solution (2 mM H_2_SO_4_). The absorbance was measured at 450 nm by a microplate reader (Varioskan Flash, Thermo Fisher Scientific, Waltham, USA). In our study, the plasma of a broiler from the PRO group in the high-oxygen room was randomly chosen as a reference. The concentration of the undiluted plasma was considered as 1,000 (arbitrary units). A standard curve was constructed from the serially double diluted plasma sample. The levels of anti-BSA IgG in all samples were calculated and compared. Negative control plasma samples (plasma of chickens which were not immunized with BSA) were included in each plate to account for plate-to-plate variations.

### Assessment of IL-1β, IL-6, IL-10, lipopolysaccharide-induced TNF-α factor (LITAF) and IL-12 in chick plasma

Cytokines (IL-1β, IL-6, IL-10, LITAF and IL-12) were measured by the ELISA methods with specific antibodies against IL-1β, IL-6, IL-10, LITAF and IL-12 prepared in our laboratory. Briefly, plasma sample was added to each well, specific rabbit anti-chicken antibodies against IL-1β, IL-6, IL-10, LITAF and IL-12 were used as capturing antibodies. After washing, peroxidase labeled goat anti-chicken IgG was added to each well. 3,3′,5,5′-Tetramethylbenzidine was served as a color indicator, and subsequently the color reaction was stopped by a stop solution (2 mM H_2_SO_4_). The absorbance values of well plates were measured at 450 nm wavelength by a microplate reader (Varioskan Flash, Thermo Fisher Scientific, Waltham, USA). The concentrations of cytokines in plasma were determined using standard curves constructed from respective cytokine standard.

### Statistical analyses

All data from the experiment, except mortality, were subjected to a two-way ANOVA by the SPSS 13.0 software for windows (SPSS, Chicago, IL). The linear model to test the effect of treatments on response variables was as follows: *y*_*ij*_ = *μ* + oxygen_*i*_ + probiotic_*j*_ + (oxygen × probiotic)_*ij*_ + *e*_*ij*_, where y = response variable, *μ* = population mean, oxygen = main effect of oxygen levels, probiotic = main effect of oral treatment of the probiotic, oxygen × probiotic = interaction effect of oxygen levels and oral treatment of the probiotic, and e = residual error [*N* (*σ*, *μ*; 0, 1)]. Mortality was analyzed by χ^2^ test. A *P*-value of less than 0.05 was considered to be statistically significant.

## Data Availability

The datasets generated and analyzed during the current study are available from the corresponding author on reasonable request.
